# Insulin resistance assessed by estimated glucose disposal rate and risk of coronary artery calcification in middle-aged participants without diabetes undergoing health check-ups

**DOI:** 10.3389/fendo.2026.1745428

**Published:** 2026-04-02

**Authors:** Ya Huang, Rui Zhang, Ying Zhou, Dandan Li, Wenji Ni, Yanhui Wan, Tao Jin, Xiangwei Bo, Xiaolin Qi, Yong Zhong

**Affiliations:** 1 Department of Health Medicine, Jinling Hospital, Affiliated Hospital of Medical School, Nanjing University, Nanjing, China; 2 Department of Healthcare, Jinling Hospital, Affiliated Hospital of Medical School, Nanjing University, Nanjing, China

**Keywords:** association, coronary artery calcification, estimated glucose disposal rate, insulin resistance, severity

## Abstract

**Background and aims:**

Insulin resistance, assessed by estimated glucose disposal rate (eGDR), is linked to atherosclerosis, yet evidence primarily comes from diabetic populations. The aim of this study was to investigate the association between eGDR and coronary artery calcification (CAC) in non-diabetic adults.

**Methods:**

In this cross-sectional study, 4750 participants aged 40–65 years without diabetes were enrolled from Jinling Hospital between 2022 and 2024. CAC was assessed via CT scans. Multivariable Logistic regression and restricted cubic splines were used to analyze the relationship of eGDR with CAC prevalence and severity.

**Results:**

The mean age was 47.98 ± 6.61 years; 79.31% were male, and 665 participants (14.0%) had CAC. After multivariable adjustment, lower eGDR was significantly associated with higher CAC risk. Each 1-unit increase in eGDR was associated with a 9% risk reduction (OR = 0.91, 95% CI: 0.84–0.98, *P* = 0.02). Compared to the lowest eGDR quartile (Q1), the odds ratios for Q2, Q3, and Q4 were 0.84 (0.66–1.07), 0.69 (0.52–0.92), and 0.63 (0.44–0.90), respectively (*P* for trend = 0.004). Tobit regression confirmed an inverse association between eGDR and CACS (β = -4.07, *P* = 0.0002). Multivariate ordered Logistic regression analysis revealed that after adjusting for multiple factors, eGDR was significantly associated with the severity of CAC, both as a continuous variable (mild CAC: OR = 0.90, 0.83–0.97; moderate-to-severe CAC: OR = 0.86, 0.78–0.95) and across quartiles (*P* for trend = 0.03). A nonlinear dose-response relationship was observed (*P* for nonlinearity = 0.01), with CAC risk increasing as eGDR fell below 10.8.

**Conclusion:**

Lower eGDR is independently associated with increased prevalence and severity of CAC in non-diabetic middle-aged Chinese adults.

## Introduction

Atherosclerosis represents a major contributor to global morbidity and mortality, responsible for millions of cardiovascular disease (CVD)-related deaths each year (1). In China, an estimated 330 million people are affected by CVD, and its prevalence continues to rise, imposing a substantial public health burden (2). As the primary pathological basis of ischemic heart disease, atherosclerosis requires early detection of subclinical stages for effective prevention. Coronary artery calcification (CAC), a hallmark of advanced atherosclerosis, serves as a crucial biomarker for evaluating both the presence and severity of coronary artery disease (3). Multislice computed tomography (CT) provides a rapid and noninvasive imaging modality, recognized for its reliability, high sensitivity, and specificity in diagnosing CAC (4). This technique enables the quantification of CAC scores, which reflect the overall burden of coronary plaque. Importantly, elevated CAC scores have been shown to independently and incrementally predict future coronary events and clinical outcomes (5).

Insulin resistance (IR), a pathophysiological condition characterized by diminished sensitivity of target tissues to insulin, impairs glucose utilization and is a well-established risk factor for atherosclerosis (6). Given the adverse implications of IR, several methods have been developed to assess it. Although the hyperinsulinemic-euglycemic clamp is considered the gold standard for identifying IR (7), its clinical utility and feasibility in large-scale epidemiological studies are limited due to the time-consuming and labor-intensive nature of the procedure. Similarly, the Homeostasis Model Assessment of Insulin Resistance (HOMA-IR) is less suitable for large population-based cohorts because of its cost and operational complexity (8). As a result, the estimated glucose disposal rate (eGDR)—a measure derived from waist circumference (WC), hypertension status, and glycated hemoglobin (HbA1c)—has emerged as a reliable surrogate marker of IR (9). This method has demonstrated high accuracy when compared with the hyperinsulinemic-euglycemic clamp technique, making it a valuable tool for assessing insulin resistance in large patient populations (10).

Recent studies have utilized eGDR as a surrogate for insulin resistance in predicting stroke, coronary artery disease, and all-cause mortality (11–13). However, most of these studies have focused primarily on individuals with diabetes, which may overestimate or confound the role of IR. Previous research consistently indicates significant heterogeneity between diabetic and non-diabetic populations (14). Individuals with diabetes are more likely to develop additional health complications, face higher cardiovascular risk, and experience increased mortality. As also highlighted by Ren et al., the non-diabetic population exhibits greater sensitivity to eGDR (15). Therefore, the objective of this study is to investigate the association between eGDR and the prevalence and severity of CAC in a large sample of non-diabetic adults undergoing routine health examinations.

## Methods

### Study design and population

This cross-sectional study included individuals aged 40 to 65 years who underwent health check-ups at the Department of Health Medicine, Jinling Hospital, Affiliated with Nanjing University, between January 2022 and December 2024, and who completed chest computed tomography (CT) examinations. For participants with multiple visits during this period, only the most recent record was included, resulting in an initial cohort of 6,788 subjects. All participants were recruited from various organizations across Jiangsu Province, representing diverse socioeconomic backgrounds.

**Figure 1:**
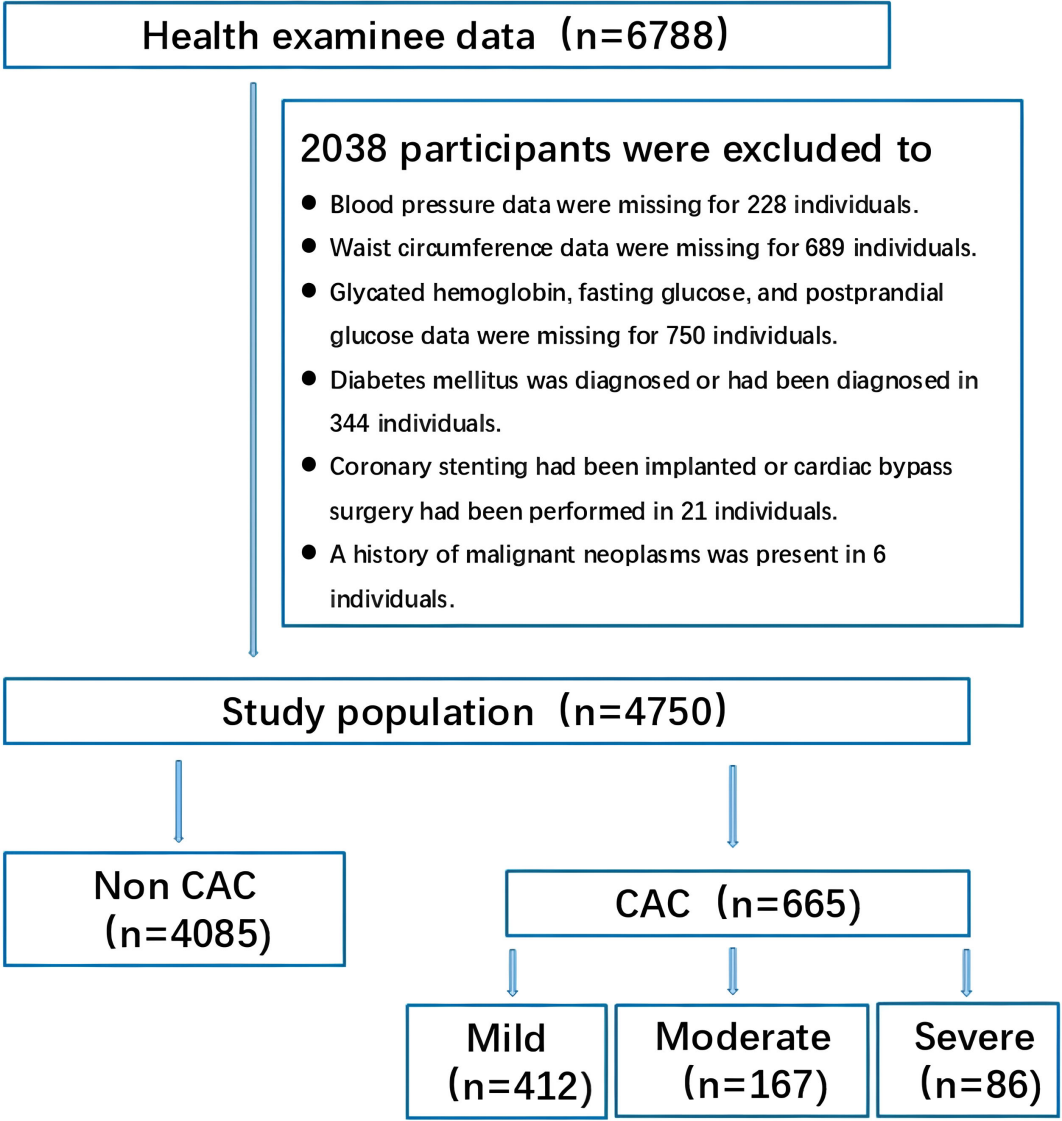
Flow chart of study participants.

As part of the check-up process, each subject was interviewed by an internist regarding lifestyle, medical history, and medication use. Anthropometric measurements and blood samples were collected by trained nurses. Laboratory analyses were performed uniformly by specialized technicians in the Laboratory Department under standardized quality control protocols. The following exclusion criteria were applied: (1) Missing blood pressure data (n = 228); (2) Missing waist circumference data (n = 689); (3) Missing glycated hemoglobin (HbA1c), fasting blood glucose (FBG), or postprandial blood glucose (PBG) data (n = 750); (4) Diagnosis of diabetes mellitus (n = 344); (5) History of coronary stenting or coronary artery bypass grafting (n = 21); (6) History of malignant neoplasms (n = 6). After applying these criteria, a total of 4,750 participants were included in the final analysis, as illustrated in **Figure 1**. The study protocol was approved by the Institutional Review Board of Jinling Hospital, Affiliated Hospital of Medical School, Nanjing University (No. 2024DZKY-015-01), and all participants provided written informed consent.

### Data collection and definition

The physician collected detailed information such as smoking and drinking habits, disease history, and medication use from the subjects during the internal medicine physical examination. Smoking status was defined as current smoking (yes/no), and drinking status was defined as current drinking (yes/no), both based on self-reported verbal inquiry during the clinical interview. Height and weight were measured using an SH-200G meter. Body mass index (BMI) was calculated by dividing weight in kilograms by height in metres squared. WC was measured by professional nurses. After a 10-minute rest period, blood pressure was measured in the non-dominant arm using an Omron sphygmomanometer. Venous blood samples were collected from subjects who had fasted for at least eight hours. FBG, triglyceride (TG), total cholesterol (TC), high-density lipoprotein cholesterol (HDL-c), low-density lipoprotein cholesterol (LDL-c), serum creatinine (SCR) and uric acid (UA) levels were measured using a Hitachi 7600 fully automated biochemistry analyzer. Venous blood was drawn two hours after breakfast to measure PBG. HbA1c levels were measured using ion exchange high-pressure liquid chromatography. Quality control of laboratory testing was performed in accordance with the Indicators of Medical Quality Control for Clinical Laboratory Specialties (2015 edition) of the National Health Commission of the People’s Republic of China.

Hypertension was defined as follows: self-reported hypertension based on physician diagnosis, and/or any use of antihypertensive medications, and/or SBP higher than 140 mmHg and/or DBP higher than 90 mmHg (16). Diabetes was defined based on a self-reported physician diagnosis, use of hypoglycemic drugs, or FBG higher than 7.0 mmol/L, or PBG higher than 11.0 mmol/L (17). Estimated glomerular filtration rate (eGFR) was calculated using the modified MDRD equation: eGFR(ml/min/1.73m^2^) =186 × (SCR) ^^-1.154^ × (age)^^-0.203^ × (0.742 female) × (1.233 Chinese) (18). The formula for calculating eGDR was as follows: eGDR (mg/kg/min) = 21.158 − (0.09 × WC) − (3.407 × hypertension) − (0.551 × HbA1c) [WC (cm), hypertension (yes = 1/no = 0), and HbA1c (%)] (19).

### Assessment of CAC

Each participant underwent a chest CT scan using a 64-slice multi-slice CT machine (SIEMENS SOMATOM Definition Flash). CAC scores (CACS) were calculated using an automated software program and the Agatston scoring method (20). Participants were categorized as follows based on the CAC scores: no CAC, CACS = 0; mild CAC, 0<CACS ≤ 100;moderate CAC, 100<CACS ≤ 300; severe CAC, CACS>300 (21).

### Statistical analysis

We used SAS software, version 9.4 (SAS Institute, Cary, NC) for statistical analysis. A two-sided *P* value of less than 0.05 was considered statistically significant. Study participants were categorized into four groups (Q1–Q4) according to quartiles of the eGDR. Continuous variables with normal distribution are presented as mean ± standard deviation (SD), while those with non-normal distribution are summarized as median (interquartile range) [M (Q1, Q3)]. Categorical variables are expressed as numbers and percentages. Trends across eGDR quartiles were assessed using linear regression for continuous variables and the Cochran–Armitage trend χ² test for categorical variables. Logistic regression was used to analyze the odds ratios (ORs) of the relationship between eGDR (continuous as well as categorical variables) and CAC. Multivariable Tobit regression models with left-censoring at zero were employed to examine factors associated with CACS, given the highly left-censored distribution of CACS. Multivariate ordered Logistic regression analysis was used to analyze the correlation between eGDR (continuous variable and categorical variable) and the severity of CAC. To allow for more flexibility in the model and visualize dose-response relationships, restricted cubic spline models with four knots at the 5th, 35th, 65th, and 95th percentiles of eGDR were constructed.To address potential confounding by smoking and drinking status, sensitivity analyses were performed with additional adjustment for these variables in the subset with available data (n = 3,360). Given the male predominance in our study (79.31%), sex-stratified analyses were conducted, and interaction terms (eGDR × sex) were included in the models to assess effect modification by sex. Multicollinearity among independent variables was assessed using variance inflation factors (VIF), with VIF < 5 considered indicative of no serious collinearity. *Post-hoc* power analyses were performed based on the observed sample sizes, event rates, and effect sizes to evaluate the statistical adequacy of our study, with power ≥80% considered adequate. Additionally, to validate the use of eGDR in non-diabetic populations, we conducted a sensitivity analysis using the triglyceride-glucose (TyG) index, calculated as ln[fasting triglycerides (mg/dL) × fasting glucose (mg/dL)/2], as an alternative insulin resistance surrogate.

## Results

### Participants characteristics

**Table 1** presents the baseline characteristics of the study participants stratified by eGDR quartiles (Q1: 8.26 ± 0.40; Q2: 10.06 ± 0.18; Q3: 10.61 ± 0.17; Q4: 11.50 ± 0.46). A total of 4750 subjects (mean age: 47.98 ± 6.61 years) with 79.31% male were included in this study. The mean age, proportion of male, SBP, DBP, BMI, WC, levels of FBG, PBG, HbA1c, TC, TG, LDL-c and UA all decreased with increasing eGDR (all *P* for trends < 0.05). However, individuals with higher levels of eGDR tended to have higher HDL-c and eGFR (*P* for trend < 0.0001). A significant inverse relationship was observed between eGDR and the prevalence of CAC, with CAC prevalence decreasing across ascending eGDR quartiles. Furthermore, higher eGDR levels were associated with an increased proportion of mild CAC and a decreased proportion of severe CAC. The trend in CAC severity across eGDR quartiles was statistically significant (*P* for trend < 0.0001).

**Table 1:** Baseline characteristics of participants stratified by quartiles of estimated glucose disposal rate.

Characteristics	Overall	Quartiles of eGDR				
		Quartile 1	Quartile 2	Quartile 3	Quartile 4	*P* for trend
n	4750	1183	1202	1174	1191	
eGDR	10.11± 0.40	8.26 ± 0.40	10.06 ± 0.18	10.61 ± 0.17	11.50 ± 0.46	< 0.0001
Age, years	47.98 ± 6.61	50.16 ± 7.33	47.87 ± 6.21	46.90 ± 6.03	46.98 ± 6.27	< 0.0001
Male, n (%)	3767 (79.31)	1043 (88.17)	1138 (94.68)	1016 (86.54)	570 (47.86)	< 0.0001
SBP, mmHg	120.56 ± 13.43	130.31 ± 14.99	119.69 ± 10.48	117.51 ± 10.81	114.74 ± 11.49	< 0.0001
DBP, mmHg	74.26 ± 9.67	80.39 ± 10.42	74.15 ± 8.09	72.33 ± 8.18	70.18 ± 8.70	< 0.0001
BMI, kg/m^2^	24.45 ± 2.65	26.58 ± 2.62	25.29 ± 1.76	23.93 ± 1.73	22.01 ± 1.93	< 0.0001
WC, cm	84.43 ± 8.81	92.43 ± 8.44	88.55 ± 2.56	83.01 ± 2.67	73.73 ± 5.32	< 0.0001
FBG, mmol/L	5.25 ± 0.49	5.46 ± 0.53	5.29 ± 0.48	5.18 ± 0.42	5.06 ± 0.42	< 0.0001
PBG, mmol/L	6.21 ± 1.33	6.76 ± 1.46	6.34 ± 1.27	6.03 ± 1.20	5.71 ± 1.14	< 0.0001
HbA1c, %	5.63 ± 0.36	5.78 ± 0.42	5.68 ± 0.31	5.58 ± 0.33	5.49 ± 0.30	< 0.0001
TC, mmol/L	5.10 ± 0.91	5.15 ± 1.00	5.10 ± 0.90	5.07 ± 0.89	5.07 ± 0.85	0.02
TG, mmol/L	1.24 (0.87 − 1.77)	1.57 (1.09 − 2.27)	1.38 (1.00 − 1.94)	1.18 (0.85− 1.64)	0.94 (0.71 − 1.32)	< 0.0001
HDL-c, mmol/L	1.28 ± 0.30	1.19 ± 0.27	1.19 ± 0.24	1.29 ± 0.28	1.44 ± 0.32	< 0.0001
LDL-c, mmol/L	2.91 ± 0.71	2.98 ± 0.78	2.98 ± 0.70	2.90 ± 0.69	2.77 ± 0.66	< 0.0001
UA, umol/L	367.59 ± 84.36	393.64 ± 84.37	391.18 ± 76.37	368.84 ± 77.79	316.68 ± 75.14	< 0.0001
eGFR, mL/min/1.73m^2^	124.02 ± 20.19	122.19 ± 21.40	122.94 ± 19.09	124.01 ± 19.12	126.94 ± 20.74	< 0.0001
Smoking status						0.27
Yes, n (%)	1252 (26.36%)	259 (21.89%)	363 (30.20%)	345 (29.39%)	285 (23.93%)	
No, n (%)	2108 (44.38%)	475 (40.16%)	543 (45.17%)	550 (46.85%)	540 (45.34%)	
Missing, n	1390 (29.26%)	449 (37.95%)	296 (24.63%)	279 (23.76%)	366 (30.73%)	
Drink status						< 0.0001
Yes, n (%)	1207 (25.41%)	320 (27.05%)	372 (30.95%)	333 (28.36%)	175 (14.69%)	
No, n (%)	2153 (45.32%)	414 (35.00%)	534 (44.42%)	562 (47.87%)	650 (54.58%)	
Missing, n	1390 (29.26%)	449 (37.95%)	296 (24.63%)	279 (23.76%)	366 (30.73%)	
CAC, n (%)	665 (14.00%)	277 (23.42%)	186 (15.47%)	123 (10.48%)	79 (6.63%)	< 0.0001
Mild, n (%)	412 (61.96%)	159 (57.40%)	110 (59.14%)	91 (73.98%)	52 (65.82%)	< 0.0001
Moderate, n (%)	167 (25.11%)	78 (28.16%)	52 (27.96%)	17 (13.82%)	20 (25.32%)	
Severe, n (%)	86 (12.93%)	40 (14.44%)	24 (12.90%)	15 (12.20%)	7 (8.86%)	

*BMI*, body mass index; *SBP*, systolic blood pressure; *DBP*, diastolic blood pressure; *eGDR*, estimated glucose disposal rate; *FBG*, fasting blood glucose; *PBG*, 2-hour postprandial blood glucose*; HbA1c*, glycosylated haemoglobin; *HDL-c*, high density lipoprotein cholesterol; *LDL-c*, low density lipoprotein cholesterol; *TC*, total cholesterol; *TG*, triglycerides; *UA*, uric acid; *WC*, waist circumference; *eGFR*, estimated glomerular filtration rate; *CAC*, coronary artery calcification.

### Association of eGDR and risk of CAC

As shown in **Table 2**, each 1-unit increase in eGDR was associated with a 17% reduction in the risk of CAC after adjustment for age and sex (OR = 0.83, 95% CI: 0.78–0.88, *P* < 0.0001). This association, though attenuated, remained statistically significant after further adjustment for body mass index, blood pressure, blood lipid profiles, uric acid, blood glucose, and eGFR (OR = 0.91, 95% CI: 0.84–0.98, *P* = 0.02).

**Table 2:** Association of eGDR with risk of CAC.

eGDR	No. of CAC/total N	Model 1		Model 2	
		OR (95%CI)	*P* value	OR (95%CI)	*P* value
Continuous					
eGDR, per 1unit	665/4750	0.83 (0.78-0.88)	< 0.0001	0.91 (0.84-0.98)	0.02
Categorical					
Q1	277/1183	ref	< 0.0001	ref	0.004
Q2	187/1202	0.71 (0.57-0.88)		0.84 (0.66-1.07)	
Q3	123/1174	0.53 (0.42-0.67)		0.69 (0.52-0.92)	
Q4	78/1191	0.44 (0.33-0.58)		0.63 (0.44-0.90)	

Model 1, adjusted for age and sex.

Model 2, additionally adjusted for body mass index, systolic blood pressure, diastolic blood pressure, total cholesterol, triglyceride, LDL-c, HDL-c, uric acid, eGFR, fasting blood glucose and 2h plasma glucose based on model 1.

Similar results were observed in the quartile-based analysis. Compared with the lowest eGDR quartile (Q1), the adjusted risks of CAC in Q2, Q3, and Q4 were significantly lower after controlling for age and sex, with reductions of 29% (OR = 0.71, 95% CI: 0.57–0.88), 47% (OR = 0.53, 95% CI: 0.42–0.67), and 56% (OR = 0.44, 95% CI: 0.33–0.58), respectively (*P* for trend < 0.0001). After additional adjustment for the full set of covariates, the association was attenuated but remained significant across eGDR quartiles (*P* for trend = 0.004).

### Association of eGDR with severity of CAC

As presented in **Table 3**, Tobit regression (accounting for 86% zero values) was used to evaluate associations with CACS. In a model adjusted for all available covariates (Model 1), eGDR was significantly and inversely associated with CACS (β = -3.70, *P* = 0.004). After further adjustment for age, sex, BMI, and fasting blood glucose (Model 2), eGDR remained a significant negative predictor of CACS (β = -4.07, *P* = 0.0002), indicating that lower eGDR is associated with higher CACS. The significant scale parameter (Sigma) in both models (*P* < 0.0001) confirmed the appropriateness of the Tobit model.

**Table 3:** Multivariable Tobit regression analysis for factors associated with CACS. .

Characteristic	Model 1			Model 2		
	β	SE	*P* value	β	SE	*P* value
eGDR	-3.70	1.27	0.004	-4.07	1.10	0.0002
age	1.94	0.21	< 0.0001	2.01	0.20	< 0.0001
sex	-14.74	3.65	< 0.0001	-13.18	3.25	< 0.0001
BMI	1.50	0.60	0.01	1.18	0.57	0.04
SBP	0.17	0.14	0.21			
DBP	-0.17	0.18	0.35			
TG	0.60	1.79	0.74			
TC	-2.86	5.02	0.57			
LDL	-2.98	5.80	0.60			
HDL	8.41	7.42	0.26			
FBG	5.65	2.88	0.0499	6.81	2.63	0.01
PBG	0.99	1.04	0.34			
HbA1c	3.63	3.86	0.35			
UA	-0.004	0.02	0.82			
eGFR	0.05	0.06	0.41			
Model statistics						
Sigma (Scale Parameter)	83.37	0.86	< 0.0001	83.54	0.86	< 0.0001

*BMI*, body mass index; *SBP*, systolic blood pressure; *DBP*, diastolic blood pressure; *eGDR*, estimated glucose disposal rate; *FBG*, fasting blood glucose; *PBG*, 2-hour postprandial blood glucose*; HbA1c*, glycosylated haemoglobin; *HDL-c*, high density lipoprotein cholesterol; *LDL-c*, low density lipoprotein cholesterol; *TC*, total cholesterol; *TG*, triglycerides; *UA*, uric acid; *WC*, waist circumference; *eGFR*, estimated glomerular filtration rate. Data were presented as coefficient (β) and standard error (SE) from Tobit regression models with left-censoring at zero to account for the 86% zero values. Model 1 includes all available covariates as listed. Model 2 retains variables that were significantly associated with CACS (P < 0.05) in Model 1, except where theoretical considerations apply.

The association between eGDR as a continuous variable and CAC severity was presented in **Table 4**. After adjustment for age and sex, each 1-unit increase in eGDR was associated with 18% (OR = 0.82, 95% CI: 0.78–0.87, *P* < 0.0001) and 21% (OR = 0.79, 95% CI: 0.73–0.86, *P* < 0.0001) reduced risks of mild CAC and moderate-to-severe CAC, respectively. Following further multivariable adjustment for BMI, blood pressure, blood lipids, eGFR, uric acid, FBG, and PBG, the associations, though slightly attenuated, remained statistically significant, with corresponding ORs of 0.90 (95% CI: 0.83–0.97, *P* = 0.007) for mild CAC and 0.86 (95% CI: 0.78–0.95, *P* = 0.003) for moderate-to-severe CAC. The point estimates suggest a slightly stronger inverse association for moderate-to-severe CAC than for mild CAC, though the confidence intervals overlapped considerably.

**Table 4:** Ordered Logistic regression of eGDR with severity of CAC.

Outcome	Model 1		Model 2	
	OR (95%CI)	*P* value	OR (95%CI)	*P* value
No CAC	ref		ref	
Mild CAC	0.82 (0.78-0.87)	< 0.0001	0.90 (0.83-0.97)	0.007
Moderate-to-severe CAC	0.79 (0.73-0.86)	< 0.0001	0.86 (0.78-0.95)	0.003

Model 1, adjusted for age and sex.

Model 2, additionally adjusted for body mass index, systolic blood pressure, diastolic blood pressure, total cholesterol, triglyceride, LDL-c, HDL-c, uric acid, eGFR, fasting blood glucose and 2h plasma glucose based on model 1.

**Table 5** showed the association between categorical eGDR and CAC severity. After adjusting for age and sex (Model 1), a significant inverse dose–response relationship was observed across eGDR quartiles (*P* for trend = 0.0002). Compared with the lowest quartile (Q1), the risk of more severe CAC was reduced by 30% in Q2 (OR = 0.70, 95% CI: 0.56–0.86; *P* = 0.0009), by 49% in Q3 (OR = 0.51, 95% CI: 0.40–0.65; *P* < 0.0001), and by 56% in Q4 (OR = 0.44, 95% CI: 0.33–0.58; *P* < 0.0001). After additional adjustment for metabolic indicators (Model 2), the inverse trend remained significant (*P* for trend = 0.03). Risk reductions persisted in Q3 (OR = 0.68, 95% CI: 0.51–0.90; *P* = 0.007) and Q4 (OR = 0.64, 95% CI: 0.45–0.92; *P* = 0.01), corresponding to reductions of 32% and 36%, respectively, while the association in Q2 was attenuated and no longer significant (OR = 0.84, 95% CI: 0.66–1.06; *P* = 0.13).

**Table 5:** Ordered Logistic regression of eGDR quartiles with severity of CAC.

eGDR	Model 1			Model 2		
	OR (95%CI)	*P* value	*P* for trend	OR (95%CI)	*P* value	*P* for trend
Q1	ref		0.0002	ref		0.03
Q2	0.70 (0.56-0.86)	0.0009		0.84 (0.66-1.06)	0.13	
Q3	0.51 (0.40-0.65)	< 0.0001		0.68 (0.51-0.90)	0.007	
Q4	0.44 (0.33-0.58)	< 0.0001		0.64 (0.45-0.92)	0.01	

Model 1, adjusted for age and sex.

Model 2, additionally adjusted for body mass index, systolic blood pressure, diastolic blood pressure, total cholesterol, triglyceride, LDL-c, HDL-c, uric acid, eGFR, fasting blood glucose and 2h plasma glucose based on model 1.

### Dose-response analysis of eGDR with CAC

**Figure 2:**
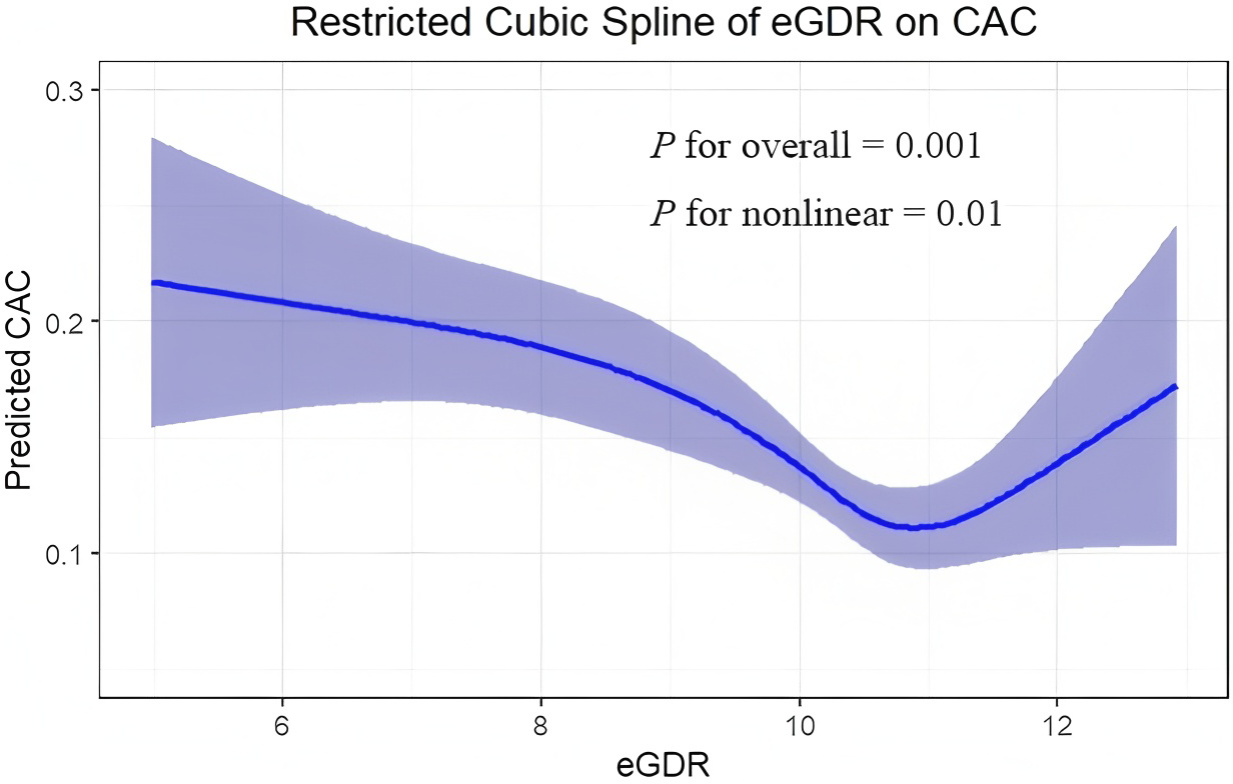
Exposure-response curves for the association between eGDR and prevalence CAC.

**Figure 2** illustrates a non-linear relationship between eGDR and the prevalence of CAC after adjusting for age, sex, BMI, blood pressure, blood lipids, eGFR, uric acid, FBG, and PBG (*P* for overall association = 0.001; *P* for non-linearity = 0.01). The dose-response curve shows that when eGDR levels fall below 10.8, the risk of CAC progressively increases with decreasing eGDR. Notably, an upturn in risk was also observed at eGDR values exceeding approximately 12, suggesting a potential J-shaped relationship, although the estimates in this range were less precise as reflected by wider confidence intervals.

### Sensitivity and supplementary analyses

To test the robustness of the association between eGDR and CAC, we conducted several sensitivity and supllementary analyses.

First, in the subset with available smoking and drinking data (n = 3360), after additional adjustment for smoking and drinking status (Model 2), the inverse association between eGDR and CAC risk remained significant. Each 1-unit increase in eGDR was associated with an 11% lower risk of CAC (OR = 0.89, 95% CI: 0.80–0.99, *P* = 0.04). Compared with participants in the lowest quartile (Q1) of eGDR, those in the highest quartile (Q4) had a 46% lower risk of CAC (OR = 0.54, 95% CI: 0.34–0.85, *P* for trend = 0.003; **Supplementary Table 1**). In the analysis of CAC severity, eGDR was also significantly associated with a lower risk of both mild CAC (OR = 0.88, 95% CI: 0.79–0.97, *P* = 0.01) and moderate-to-severe CAC (OR = 0.83, 95% CI: 0.73–0.95, *P* = 0.007) after full adjustment (**Supplementary Table 2**). Consistently, participants in the higher eGDR quartiles showed significantly lower odds of more severe CAC (*P* for trend = 0.02; **Supplementary Table 3**).

Second, we examined the TyG index in a sensitivity analysis. Although higher TyG levels were significantly associated with increased CAC risk in the age- and sex-adjusted model (OR = 1.22, 95% CI: 1.05–1.42, *P* = 0.01), this association was attenuated and became non-significant after further adjustment for metabolic factors (Model 2: OR = 1.07, 95% CI: 0.84–1.36, *P* = 0.59). A similar pattern was observed in the quartile-based analysis, where the significant risk observed in Q4 in Model 1 (OR = 1.48, 95% CI: 1.15–1.91) was no longer significant after full adjustment (**Supplementary Table 4**).

Third, in sex-stratified analyses, the inverse association between eGDR and CAC was statistically significant in both males (OR = 0.88, 95% CI: 0.81–0.95, *P* = 0.002) and females (OR = 0.83, 95% CI 0.69–0.99, *P* = 0.03). No significant interaction was observed between eGDR and sex (*P* for interaction = 0.64 for continuous; **Supplementary Table 5**).

Fourth, multicollinearity assessment using variance inflation factors indicated that the association between eGDR and CAC was not biased by multicollinearity. Despite high collinearity between total cholesterol (VIF = 14.31) and LDL-c (VIF = 11.56), the VIF for eGDR was acceptably low (2.11), confirming the robustness of our primary findings (**Supplementary Table 6**).

Finally, a *post-hoc* power analysis confirmed that our study was adequately powered to detect the observed associations. As showed in **Supplementary Table 7**, for the primary analysis of CAC presence, the power exceeded 99% for the continuous eGDR analysis (per 1-unit increase) and was 86.2% for the comparison between the highest and lowest eGDR quartiles. For the severity analysis using ordered logistic regression, the power was 91.5% for the continuous analysis and 84.7% for the quartile comparison. These results indicate that the sample size was sufficient to detect the observed effect sizes with acceptable statistical power.

## Discussion

This study demonstrates that a lower eGDR, a surrogate for IR, is significantly associated with an increased risk and severity of CAC in non-diabetic, middle-aged adults. These associations were independent of traditional cardiovascular risk factors, suggesting that IR contributes to coronary atherosclerosis independently of conventional metabolic abnormalities.

This association can be explained by the biological role of IR in atherogenesis. Insulin resistance—characterized by an impaired cellular response to insulin in adipose, muscle, and hepatic tissues—represents a fundamental pathophysiological defect that precedes and predicts type 2 diabetes mellitus (T2DM) by years or decades (22, 23). To compensate for peripheral IR, pancreatic β-cells enhance insulin secretion, resulting in a state of compensatory hyperinsulinemia (24). This hyperinsulinemic state directly promotes atherosclerosis through multiple interconnected mechanisms: (i) stimulating *de novo* lipogenesis and increasing very low-density lipoprotein secretion (25); (ii) enhancing vascular smooth muscle cell proliferation and migration (26); (iii) activating pro-inflammatory gene expression (27); (iv) increasing collagen synthesis (28); and (v) facilitating LDL-C transport into arterial wall cells (29). Critically, because these proatherogenic processes operate independently of hyperglycemia (30), IR likely initiates vascular injury long before the clinical onset of diabetes—underscoring the importance of early risk assessment in non-diabetic populations.

The eGDR offers a practical approach to quantifying IR using three routinely available clinical parameters: WC, hypertension status, and HbA1c—all established cardiovascular risk factors in their own right. Initially developed in Western diabetic cohorts (9), eGDR has been widely applied to non-diabetic and multi-ethnic populations (30). Recent studies in Chinese non-diabetic cohorts have identified eGDR as a potential predictor and intervention target for cardiovascular disease (31), with multi-ethnic atherosclerotic research confirming a linear inverse relationship between eGDR and atherosclerotic CVD risk (32). Our findings extend this evidence by demonstrating that eGDR is independently associated with subclinical coronary atherosclerosis, as measured by CAC, in non-diabetic individuals. Thus, eGDR may represent a practical, non-genetic marker for cardiovascular risk assessment.

### Practical implications for routine health check-ups

The findings have direct implications for cardiovascular risk stratification in routine clinical settings. eGDR offers several advantages for integration into annual health examinations. Its three components—WC, blood pressure, and HbA1c—are already collected during standard check-ups, requiring no additional tests or costs. Unlike insulin-based indices such as HOMA-IR, eGDR avoids pre-analytical challenges including sample instability and the need for fasting samples. Additionally, eGDR provides a single numeric value that can be automatically calculated and included on routine laboratory reports.

For healthcare providers, a low eGDR value could prompt targeted lifestyle counseling focused on reducing central obesity and managing blood pressure. For population screening, eGDR may serve as a simple, cost-effective tool to identify non-diabetic individuals who could benefit from early preventive interventions. Future research should evaluate whether incorporating eGDR into routine health check-ups improves risk stratification and ultimately reduces cardiovascular event rates.

### Comparison with other insulin resistance surrogates

The eGDR provides distinct advantages for assessing cardiovascular risk in non-diabetic populations compared to other IR surrogates. Unlike HOMA-IR, which requires insulin assays and is variable in normoglycemic states (33), eGDR dispenses with insulin measurement by using accessible clinical measures without losing predictive power. Whereas the TyG index focuses on lipid-related IR (34) and can be confounded by hypertriglyceridemia, eGDR directly incorporates the vascular risk of hypertension. Similarly, metabolic score for insulin resistance (METS-IR) includes lipids and BMI but omits blood pressure (35), a limitation eGDR avoids. Our findings suggest that eGDR’s inclusion of hypertension enables it to better capture the combined metabolic and hemodynamic insults in early atherosclerosis, solidifying its utility for CAC risk stratification in routine screenings.

Notably, our sensitivity analyses using the TyG index as an alternative insulin-free surrogate marker support the unique value of eGDR. While TyG was significantly associated with CAC in age- and sex-adjusted models, this association was substantially attenuated and became non-significant after full multivariable adjustment, whereas eGDR remained significantly associated across all models. This discrepancy likely reflects fundamental differences in what these indices capture: eGDR integrates glycemic, adiposity, and hemodynamic components, while TyG primarily reflects lipid-glucose metabolism. These findings align with recent studies that have successfully employed eGDR in non-diabetic cohorts, including Chinese adults from the CHARLS study (31) and CKD patients from the UK Biobank (36), all demonstrating its predictive value for cardiovascular outcomes beyond that of other IR surrogates.

### Non-linear relationship and methodological considerations

Restricted cubic spline analysis revealed a non-linear dose-response relationship between eGDR and CAC risk (P for nonlinearity = 0.01). While CAC risk increased progressively as eGDR decreased below approximately 10.8, an upturn in risk was observed at eGDR values exceeding 12, suggesting a potential J-shaped association. This finding warrants cautious interpretation, and several potential explanations may account for this pattern. First, extremely high eGDR values may reflect unusually low body mass index or blood pressure, which in some contexts can be associated with underlying conditions such as chronic illness, malnutrition, or frailty—factors that may paradoxically increase cardiovascular risk. Second, this could represent a statistical artifact due to sparse data at the upper end, as reflected in the wider confidence intervals. Although such J-shaped relationships have been reported for other cardiovascular risk factors (37, 38), further studies are needed to determine whether this represents a true biological phenomenon or a statistical artifact.

The eGDR formula was originally developed in type 1 diabetes (9), raising concerns about its calibration and potential overestimation of insulin sensitivity in non-diabetic populations. To address this, we conducted sensitivity analyses demonstrating the robustness of our findings: (i) the association persisted after adjusting for eGDR components; (ii) eGDR outperformed the TyG index, which was attenuated after full adjustment; and (iii) our findings align with recent non-diabetic cohort studies (31, 36). Nevertheless, without direct clamp validation in normoglycemic individuals, the precise degree of miscalibration cannot be quantified. Future studies should recalibrate eGDR for non-diabetic populations; until then, our findings demonstrate an association between a composite clinical score (WC, hypertension, HbA1c) and CAC, which likely reflects underlying insulin resistance.

### Limitations

Several limitations warrant consideration. First, as an observational cross-sectional study, causality cannot be inferred from the association between eGDR and CAC. Second, selection bias and limited generalizability are significant concerns. Our cohort consisted predominantly of men (79.31%) who voluntarily participated in health examinations from organizations in Jiangsu Province, likely representing a higher socioeconomic stratum and introducing potential healthy volunteer bias. Although sex-stratified analyses showed consistent inverse associations with non-significant interaction tests (P for interaction > 0.05), the smaller sample size and lower event rate in women limited statistical power for subgroup analyses, warranting future studies with balanced sex representation. Third, despite adjustments for known confounders, residual confounding from unmeasured or imprecisely measured factors cannot be entirely excluded. These may include socioeconomic factors (e.g., income, education, occupational physical activity), dietary patterns, and family history of cardiovascular disease, which could differ systematically in our relatively homogeneous, higher socioeconomic status sample. Fourth, data on smoking and alcohol history were obtained via verbal inquiry by physicians rather than validated questionnaires, raising concerns about potential measurement error. To prioritize model reliability, these variables were not included in the primary analyses. However, acknowledging their importance as established CVD risk factors, we performed sensitivity analyses adjusting for smoking and alcohol consumption in the subset with available data (n = 3,360). The results remained consistent, suggesting that our findings are not substantially confounded by these factors. Nevertheless, the reduced sample size and self-reported nature of these variables mean that residual confounding cannot be entirely ruled out. Despite these limitations, the consistency of our findings across multiple sensitivity analyses strengthens confidence in the robustness of the observed associations.

## Conclusion

In conclusion, this study demonstrates that lower eGDR is independently associated with increased risk and severity of CAC in non-diabetic, middle-aged Chinese adults. As a simple, non-insulin-based marker derived from routine clinical parameters, eGDR holds promise for integration into standard health check-ups to identify individuals at elevated cardiovascular risk who may benefit from early lifestyle interventions. Further prospective studies are warranted to validate these findings and establish eGDR-guided preventive strategies for reducing the population burden of cardiovascular disease, given its simplicity and accessibility as a tool for early risk stratification in non-diabetic populations.

## Data Availability

The raw data supporting the conclusions of this article will be made available by the authors, without undue reservation.

## Supplementary material

Supplementary Table 1 Sensitivity Analysis: Association of eGDR with risk of CAC after additional adjustment for smoking and drinking status. Model 1, adjusted for age and sex. Model 2, additionally adjusted for smoking and drinking status, body mass index, systolic blood pressure, diastolic blood pressure, total cholesterol, triglyceride, LDL-c, HDL-c, uric acid, eGFR, fasting blood glucose and 2h plasma glucose based on model 1.

Supplementary Table 2 Sensitivity Analysis: Multivariate ordered Logistic regression of eGDR on the severity of CAC after additional adjustment for smoking and drinking status. Model 1, adjusted for age and sex. Model 2, additionally adjusted for smoking and drinking status, body mass index, systolic blood pressure, diastolic blood pressure, total cholesterol, triglyceride, LDL-cholesterol, HDL-cholesterol, uric acid, eGFR, fasting blood glucose and 2h plasma glucose based on model 1.

Supplementary Table 3 Sensitivity Analysis: Multivariate ordered Logistic regression of the quartiles of eGDR and the severity of CAC after additional adjustment for smoking and drinking status. Model 1, adjusted for age and sex. Model 2, additionally adjusted for smoking and drinking status, body mass index, systolic blood pressure, diastolic blood pressure, total cholesterol, triglyceride, LDL-cholesterol, HDL-cholesterol, uric acid, eGFR, fasting blood glucose and 2h plasma glucose based on model 1.

Supplementary Table 4 Sensitivity Analysis: Association of TyG with risk of CAC. Model 1, adjusted for age and sex. Model 2, additionally adjusted for smoking and drinking status, body mass index, systolic blood pressure, diastolic blood pressure, total cholesterol, LDL-c, HDL-c, uric acid, eGFR based on model 1.

Supplementary Table 5 Sensitivity Analysis: Association of eGDR with risk of CAC stratified by sex. Model 1, adjusted for age. Model 2, additionally adjusted for smoke status, alcohol consumption, body mass index, systolic blood pressure, diastolic blood pressure, total cholesterol, triglyceride, LDL-c, HDL-c, uric acid, eGFR, fasting blood glucose and 2h plasma glucose based on model 1.

Supplementary Table 6 Variance inflation factors for variables in the fully adjusted model. *VIF*, variance inflation factors; *eGDR*, estimated glucose disposal rate; *BMI*, body mass index; *SBP*, systolic blood pressure; *DBP*, diastolic blood pressure; *TC*, total cholesterol; *TG*, triglycerides; *LDL-c*, low density lipoprotein cholesterol; *HDL-c*, high density lipoprotein cholesterol; *TC*, total cholesterol; *TG*, triglycerides; *UA*, uric acid; *eGFR*, estimated glomerular filtration rate; *FBG*, fasting blood glucose; *PBG*, 2-hour postprandial blood glucose.

Supplementary Table 7 *Post-hoc* power analysis for the association between eGDR and CAC. *Power calculations were performed at α = 0.05 (two-sided) based on observed sample sizes, event rates, and effect sizes from the study.
