# Supplementary material for: Insulin resistance assessed by estimated glucose disposal rate and risk of coronary artery calcification in middle-aged participants without diabetes undergoing health check-ups
Source: Front Endocrinol (Lausanne). 2026 Apr 2;17:1745428. doi: 10.3389/fendo.2026.1745428 (PMC13082989; doi:10.3389/fendo.2026.1745428)
Supplement: Supplementary Table 1 — Sensitivity Analysis: Association of eGDR with risk of CAC after additional adjustment for smoking and drinking status. Model 1, adjusted for age and sex. Model 2, additionally adjusted for smoking and drinking status, body mass index, systolic blood pressure, diastolic blood pressure, total cholesterol, triglyceride, LDL-c, HDL-c, uric acid, eGFR, fasting blood glucose and 2h plasma glucose based on model 1. [file DataSheet1.docx]

**Supplementary** **Materials**

Table S1. Sensitivity Analysis: Association of eGDR with risk of CAC after additional adjustment for smoking and drinking status.

|  | No. of CAC / total N | Model 1 | | Model 2 | |
| --- | --- | --- | --- | --- | --- |
|  |  | OR (95%CI) | *P* value | OR (95%CI) | *P* value |
| Continuous | | | | | |
| eGDR, per 1unit | 425 / 3360 | 0.81 (0.75-0.87) | < 0.0001 | 0.89 (0.80-0.99) | 0.04 |
| Categorical | | | | | |
| Q1 | 169 / 838 | ref | < 0.0001 | ref | 0.003 |
| Q2 | 130 / 846 | 0.75 (0.58-0.97) |  | 0.88 (0.66-1.16) |  |
| Q3 | 81 / 841 | 0.51 (0.38-0.69) |  | 0.65 (0.46-0.92) |  |
| Q4 | 45 / 835 | 0.38 (0.27-0.56) |  | 0.54 (0.34-0.85) |  |

Model 1, adjusted for age and sex.

Model 2, additionally adjusted for smoking and drinking status, body mass index, systolic blood pressure, diastolic blood pressure, total cholesterol, triglyceride, LDL-c, HDL-c, uric acid, eGFR, fasting blood glucose and 2h plasma glucose based on model 1.

Table S2 Sensitivity Analysis: Multivariate ordered Logistic regression of eGDR on the severity of CAC after additional adjustment for smoking and drinking status.

| Outcome | Model 1 | | Model 2 | | |
| --- | --- | --- | --- | --- | --- |
|  | OR (95%CI) | *P* value | | OR (95%CI) | *P* value |
| No CAC | ref |  | | ref |  |
| Mild CAC | 0.81 (0.75-0.87) | < 0.0001 | | 0.88 (0.79-0.97) | 0.01 |
| Moderate and severe CAC | 0.76 (0.68-0.86) | < 0.0001 | | 0.83 (0.73-0.95) | 0.007 |

Model 1, adjusted for age and sex.

Model 2, additionally adjusted for smoking and drinking status, body mass index, systolic blood pressure, diastolic blood pressure, total cholesterol, triglyceride, LDL-cholesterol, HDL-cholesterol, uric acid, eGFR, fasting blood glucose and 2h plasma glucose based on model 1.

Table S3 Sensitivity Analysis: Multivariate ordered Logistic regression of the quartiles of eGDR and the severity of CAC after additional adjustment for smoking and drinking status.

| eGDR | Model 1 | | |  | Model 2 | | |
| --- | --- | --- | --- | --- | --- | --- | --- |
|  | OR (95%CI) | *P* value | *P* for trend |  | OR (95%CI) | *P* value | *P* for trend |
| Q1 | ref |  | < 0.0001 | | ref |  | 0.02 |
| Q2 | 0.73 (0.56-0.94) | 0.02 |  |  | 0.86 (0.65-1.14) | 0.29 |  |
| Q3 | 0.50 (0.37-0.66) | <0.0001 |  |  | 0.63 (0.44-0.89) | 0.008 |  |
| Q4 | 0.39 (0.27-0.56) | <0.0001 |  |  | 0.54 (0.34-0.86) | 0.009 |  |

Model 1, adjusted for age and sex.

Model 2, additionally adjusted for smoking and drinking status, body mass index, systolic blood pressure, diastolic blood pressure, total cholesterol, triglyceride, LDL-cholesterol, HDL-cholesterol, uric acid, eGFR, fasting blood glucose and 2h plasma glucose based on model 1.

Table S4. Sensitivity Analysis: Association of TyG with risk of CAC.

|  | No. of CAC / total N | Model 1 | | Model 2 | |
| --- | --- | --- | --- | --- | --- |
|  |  | OR (95%CI) | *P* value | OR (95%CI) | *P* value |
| Continuous | | | | | |
| TyG, per 1unit | 665 / 4750 | 1.22 (1.05-1.42) | 0.01 | 1.07 (0.84-1.36) | 0.59 |
| Categorical | | | | | |
| Q1 | 169 / 838 | ref | 0.01 | ref | 0.55 |
| Q2 | 130 / 846 | 1.38 (1.07-1.80) |  | 1.29 (0.98-1.69) |  |
| Q3 | 81 / 841 | 1.27 (0.98-1.66) |  | 1.10 (0.82-1.47) |  |
| Q4 | 45 / 835 | 1.48 (1.15-1.91) |  | 1.20 (0.86-1.68) |  |

Model 1, adjusted for age and sex.

Model 2, additionally adjusted for smoking and drinking status, body mass index, systolic blood pressure, diastolic blood pressure, total cholesterol, LDL-c, HDL-c, uric acid, eGFR based on model 1.

Table S5. Sensitivity Analysis: Association of eGDR with risk of CAC stratified by sex.

|  | No. of CAC / total N | Model 1 | | Model 2 | |
| --- | --- | --- | --- | --- | --- |
|  |  | OR (95%CI) | *P* value | OR (95%CI) | *P* value |
| Male | | | | | |
| Continuous eGDR, per 1unit | 607 / 3167 | 0.83 (0.78-0.88) | < 0.0001 | 0.88 (0.81-0.95) | 0.002 |
| Categorical | | | | | |
| Q1 | 256 / 1043 | ref | < 0.0001 | ref | 0.02 |
| Q2 | 179 / 1138 | 0.70 (0.56-0.87) |  | 0.84 (0.66-1.08) |  |
| Q3 | 116 / 1016 | 0.54 (0.42-0.69) |  | 0.72 (0.53-0.98) |  |
| Q4 | 56 / 570 | 0.45 (0.33-0.62) |  | 0.66 (0.44-0.98) |  |
| Female | | | | | |
| Continuous eGDR, per 1unit | 58 / 923 | 0.82 (0.70-0.96) | 0.01 | 0.83 (0.69-0.99) | 0.03 |
| Categorical | | | | | |
| Q1 | 21 / 140 | ref | 0.01 | ref | 0.08 |
| Q2 | 8 / 64 | 0.94 (0.38-2.33) |  | 0.89 (0.31-2.59) |  |
| Q3 | 7 / 158 | 0.42 (0.17-1.04) |  | 0.43 (0.15-1.25) |  |
| Q4 | 22 / 621 | 0.44 (0.23-0.87) |  | 0.47 (0.18-1.25) |  |
| *P* for interaction | | | | | |
| Continuous eGDR × sex | | 0.54 | | 0.64 | |
| Categorical eGDR × sex | | 0.62 | | 0.75 | |

Model 1, adjusted for age.

Model 2, additionally adjusted for smoke status, alcohol consumption, body mass index, systolic blood pressure, diastolic blood pressure, total cholesterol, triglyceride, LDL-c, HDL-c, uric acid, eGFR, fasting blood glucose and 2h plasma glucose based on model 1.

Table S6 Variance inflation factors for variables in the fully adjusted model.

| Variable | VIF |
| --- | --- |
| eGDR | 2.11 |
| Age | 1.32 |
| Sex | 1.49 |
| BMI | 1.74 |
| SBP | 2.29 |
| DBP | 2.05 |
| TC | 14.31 |
| TG | 2.65 |
| LDL-c | 11.56 |
| HDL-c | 3.33 |
| UA | 1.65 |
| eGFR | 1.17 |
| FBG | 1.25 |
| PBG | 1.27 |

*VIF:* variance inflation factors, *eGDR*, estimated glucose disposal rate, *BMI*, body mass index, *SBP,* systolic blood pressure, *DBP*, diastolic blood pressure, *TC,* total cholesterol, *TG,* triglycerides, *LDL-c,* low density lipoprotein cholesterol, *HDL-c,* high density lipoprotein cholesterol, *TC,* total cholesterol, *TG,* triglycerides, *UA,* uric acid, *eGFR*, estimated glomerular filtration rate, *FBG*, fasting blood glucose, *PBG,* 2-hour postprandial blood glucose.

Table S7. Post-hoc power analysis for the association between eGDR and CAC.

| Analysis | Comparison | N | Event/Outcome | Observed OR (95% CI) | | Power (%) | |
| --- | --- | --- | --- | --- | --- | --- | --- |
| Primary analysis | | | | | | | |
| Multivariate logistic regression | Per 1-unit increase | 4,750 | CAC presence (n=665) | 0.83 (0.78 -0.88) | | > 99 | |
| Multivariate logistic regression | Q4 (n = 1,191) vs Q1 (n = 1,183) | 2374 | CAC presence (n=355) | 0.63 (0.44 -0.90) | | 86.2 | |
| Severity analysis | | | | | | | |
| Multivariate ordered logistic regression | Per 1-unit increase | 4750 | CAC severity (3 levels) | Mild: 0.90 (0.83 - 0.97 Moderate-to-severe: 0.86 (0.78 - 0.95) | | 91.5 | |
| Multivariate ordered logistic regression | Q4 (n = 1,191) vs Q1 (n = 1,183) | 2,374 | CAC severity | 0.64 (0.45–0.92) | 84.7 | |  |

*Power calculations were performed at α = 0.05 (two-sided) based on observed sample sizes, event rates, and effect sizes from the study.
